# Effects of Different Intervention Factors on Vascular Endothelial Growth Factor-Induced Human Airway Smooth Muscle Cell Migration

**DOI:** 10.1155/2022/6879539

**Published:** 2022-10-10

**Authors:** Chengtian Lv, Guangyuan Liao, Lichan Wu, Jing Li, Yuanmei Gao

**Affiliations:** Department of Critical Care Medicine, Guangdong Provincial Key Laboratory of Major Obstetric Diseases, The Third Affiliated Hospital of Guangzhou Medical University, Guangzhou, China

## Abstract

**Background:**

Asthma airway remodeling is closely related to the abnormal migration of human airway smooth muscle cells (ASMCs), and vascular endothelial growth factor (VEGF) is involved in the pathophysiological process of asthma. This study aimed to investigate the effect of VEGF on ASMC migration through *in vitro* cell experiments and to intervene in ASMC migration with different asthma drugs and signaling pathway inhibitors to provide a basis for screening effective drugs for airway remodeling.

**Methods:**

The effect of VEGF on the proliferation of ASMCs was detected by the CCK-8 method, and the effect of VEGF on the migration of ASMCs was proven by scratch and transwell assays. Different asthma drugs and signaling pathway inhibitors were used to interfere with the migration of ASMCs. The number of migrating cells was compared between the intervention and nonintervention groups.

**Results:**

Our results showed that VEGF induction enhanced ASMC migration; pretreatment with the commonly used asthma drugs (salbutamol, budesonide, and ipratropium bromide) significantly attenuated VEGF-induced ASMC migration; and inhibitors SB203580, LY294002, and Y27632 blocked the VEGF-induced activation of p38 MAPK, PI3K, and ROCK signaling pathway targets in ASMCs and inhibited migration.

**Conclusion:**

This study shows that the current commonly used asthma drugs salbutamol, budesonide, and ipratropium have potential value in the treatment of airway remodeling, and the p38 MAPK, PI3K, and ROCK signaling pathway targets are involved in the VEGF-induced ASMC migration process. Signaling pathway inhibitor drugs may be a new way to treat asthma-induced airway remodeling in asthma patients in the future. However, the related mechanism and safety profile still need further research.

## 1. Introduction

Asthma is a global public health problem; more than 300 million people worldwide suffer from asthma, and approximately 40 million people die of asthma every year [1–3]. Airway remodeling is an abnormal change in the structure and molecular weight of the airway wall, which can lead to irreversible airflow limitation and persistent nonspecific bronchial hyperresponsiveness and can reduce the lung function of patients. It is an important pathological feature of asthma development [4]. The pathogenesis of airway remodeling is multifaceted and closely related to the abnormal migration of ASMCs [5–7].

Vascular endothelial growth factor (VEGF) promotes vascular endothelial cell proliferation, inducing angiogenesis and increasing microvascular permeability [8]. The VEGF level in the bronchoalveolar lavage fluid of asthma patients is higher than that of healthy controls, whereas VEGF overexpression in induced sputum and plasma correlates with disease severity [9]. VEGF induces vascular endothelial growth, participates in chronic airway inflammation and mucosal neovascularization, and negatively correlates with tracheal lumen diameter [10]. Therefore, VEGF is involved in the pathophysiological process of asthma and plays an important role in airway remodeling.

Currently, the effect of VEGF on the migration of ASMCs is unclear, and there is still no effective drug for the treatment of asthma-related airway remodeling. To further clarify the effect of VEGF on the migration of ASMCs, we performed *in vitro* cell experiments and investigated the VEGF-induced migration of ASMCs with different drugs to explore new methods to inhibit ASMC migration.

## 2. Materials and Methods

### 2.1. Cell Source, Culture, and Identification

**Figure 1:**
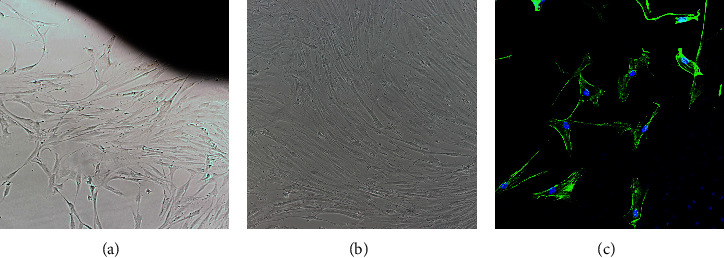
The morphology of ASMCs and ɑ-actin immunohistochemical staining were observed under an inverted microscope. (a) ASMCs grew at the edge of the tissue patch after 3–5 days of tissue patch culture. (b) ASMCs grew and merged into a “peak valley” shape. (c) Immunohistochemical staining of *α*-actin.

This study was approved by the Ethics Committee of the Third Affiliated Hospital of Guangzhou Medical University, and primary cells were obtained from tracheal smooth muscle tissue from organ donation donors. We used the tissue block adherent culture method to obtain primary ASMCs. When the cell fusion rate reached approximately 90%, the cells were subcultured. In this study, cells in the logarithmic growth phase at passages 3–6 were used for experiments. We observed the cell size, morphology, and growth characteristics under an inverted microscope. The smooth muscle cells were spindle-shaped, with one or more nucleoli located in the center of the cell, showing typical “peak-valley” features under phase contrast microscopy. The cultured cells were detected by the cell immunofluorescence method, and ɑ-smooth muscle actin (*α*-actin) indicated smooth muscle cells (Figure 1).

### 2.2. Cell Proliferation Assay

We digested ASMCs with 0.25% Trypsin-EDTA, adjusted the concentration of the cell suspension to 1 × 10^5^ cells/ml, seeded the cells into 96-well plates, and cultured them in a 5% CO_2_ incubator at 37°C for 24 h. After the cells were completely attached, the serum-containing medium was removed by aspiration and replaced with a serum-free medium, and the cells were starved for 24 h. After culturing the cells in the incubator for 24 h, the medium was removed by suction, the basal medium was added, and VEGF (Novoprotein) was added to the VEGF group at 5, 10, 20, 40, or 80 ng·ml^−1^. After 24 h, the medium was changed to a medium containing 10% CCK-8 reagent. After another 4 h of culture, the OD value was measured with a microplate reader at a wavelength of 450 nm.

### 2.3. Wound Healing Assay

Before the experiment, lines were marked on the bottom of the 6-well cell culture plate, with an interval of 0.5 cm. After ASMCs were digested with 0.25% Trypsin-EDTA, the concentration of the cell suspension was adjusted to 2 × 10^5^ cells/ml, inoculated into 6-well plates, and cultured in a 5% CO_2_ incubator at 37°C for 24 h. The medium was changed to a serum-free medium after attachment, and the cells were starved for 24 h. A scratch was made with a 200 *μ*l pipette tip perpendicular to the horizontal line on the back, a basal medium was added to the control group, and a medium containing VEGF (20 or 40 ng·ml^−1^) was added to the VEGF group. The scratches were photographed under a microscope at 0 h, placed in a cell incubator for continued culture, and then photographed under a microscope at 12 and 24 h. The cell migration rate was calculated by analyzing the changes in the scratch area.

### 2.4. Transwell Assay

We used transwells (Corning, NY, USA) with 8 *μ*m channels for cell migration experiments. The medium was changed to a serum-free medium 24 h before the experiment, and the cells were starved and placed in a 37°C, 5% CO_2_ incubator. The cells were digested with trypsin, and the concentration of the cell suspension was adjusted to 8.0 × 10^5^ cells/ml. ASMCs were added to the upper chamber, and basal medium, VEGF (20 or 40 ng·ml^−1^), and PDGF (10 ng·ml^−1^) were added to the lower chamber (the PDGF group was used as a positive control group). The cells were incubated in a cell incubator, and the migration experiment was stopped after 12 and 24 h. The cells that had migrated to the lower surface were fixed with 4% paraformaldehyde for 15 min and stained with 1% crystal violet for 30 min. Then, the migrating cells were photographed with an inverted microscope (×200 magnification) and counted using ImageJ software.

### 2.5. Effects of Asthma Drugs on ASMC Migration

We used transwell chambers (Corning, NY, USA) with an 8 *μ*m pore size to detect cell migration. The medium was changed to a serum-free medium 24 h before the experiment, and the cells were starved and placed in a 37°C, 5% CO_2_ incubator. Cells were trypsinized and centrifuged. Albuterol (Suzhou Hongsen Pharmaceutical Co., Ltd., China), budesonide (AstraZeneca Pty Ltd.), and ipratropium bromide (Boehringer Ingelheim Pharma GmbH & Co. KG) were diluted in DMEM containing 0.3% BSA. The concentration was 1.0 × 10^−8^ times that of the original solution, and the concentration of the cell suspension was adjusted to 8.0 × 10^5^ cells/ml added to the upper chamber of the Transwell. The intervention group was pretreated by adding the above asthma drugs in the upper chamber for 30 min, the control group was added with a basal medium in the lower chamber, and the experimental and intervention groups were added with VEGF (40 ng·ml^−1^) in the lower chamber. After 24 h of incubation at 37°C, the cells that had migrated to the lower surface were fixed with 4% paraformaldehyde for 15 min and stained with 1% crystal violet for 30 min. Then, the migrating cells were photographed with an inverted microscope (×200 magnification) and counted using ImageJ software.

### 2.6. Effects of Signaling Pathway Inhibitors on ASMC Migration

We used transwell chambers (Corning, NY, USA) with a pore size of 8 *μ*m to detect cell migration. In total, 24 h before the experiment, the medium was changed to a serum-free medium, and the cells were starved and placed in a 37°C, 5% CO_2_ incubator. The cells were trypsinized and centrifuged. SB203580 (MedChemExpress), Y27632 (MedChemExpress), and LY294002 (MedChemExpress) were diluted in DMEM containing 0.3% BSA at a concentration of 25 nM/ml for SB203580, 10 nM/ml for Y27632, and 10 nM/ml for LY294002. The concentration of the cell suspension was adjusted to 8.0 × 10^5^ cells/ml added to the upper chamber of the Transwell. The intervention group was pretreated by adding the above signal pathway inhibitors in the upper chamber for 30 min, the control group was added with a basal medium in the lower chamber, and the experimental and intervention groups were added with VEGF (40 ng·ml^−1^) in the lower chamber. After 24 h of incubation at 37°C, the cells that had migrated to the lower surface were fixed with 4% paraformaldehyde for 15 min and stained with 1% crystal violet for 30 min. Then, the migrating cells were photographed with an inverted microscope (×200 magnification) and counted using ImageJ software.

### 2.7. Statistical Analysis

Statistical analysis was performed using SPSS 25.0 software (SPSS Inc., Chicago, IL). The data conformed to a normal distribution and were expressed as the mean ± standard deviation. One-way ANOVA was used to determine statistical significance in the mean variance of multiple groups, the LSD method was used for pairwise comparison, and Dunnett's T3 method was used to assess the heterogeneity of variance. When *P* < 0.05, the difference was considered statistically significant.

## 3. Results

### 3.1. CCK-8 Assay to Detect the Effects of VEGF on ASMC Proliferation

**Figure 2:**
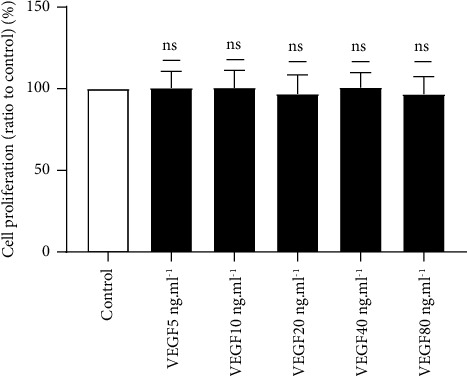
A CCK-8 assay was used to determine the effects of VEGF on ASMC proliferation. The 5 and −80 ng·ml^−1^ concentration ranges showed no significant effect on ASMC proliferation activity (F = 0.713, *P* > 0.05). NS indicates no significant difference.

We used CCK-8 assays to detect the effect of VEGF on ASMC proliferation. The cell proliferation of the VEGF groups (5–80 ng·ml^−1^) was different from that of the control group. With the control group value set to 1, the VEGF 5 ng·ml^−1^ group value was 1.0047 ± 0.1008, the VEGF 10 ng·ml^−1^ group value was 1.0054 ± 0.1072, the VEGF 20 ng·ml^−1^ group value was 0.9681 ± 0.1174, the VEGF 40 ng·ml^−1^ group values was 1.0067 ± 0.0914, and the VEGF 80 ng·ml^−1^ group value was 0.9669 ± 0.1068. The results showed no significant difference in cell proliferation between the different concentrations of the VEGF and control groups (*F* = 0.713, *P* > 0.05) (Figure 2).

### 3.2. Wound Healing Assay to Detect VEGF-Induced Migration of ASMCs

**Figure 3:**
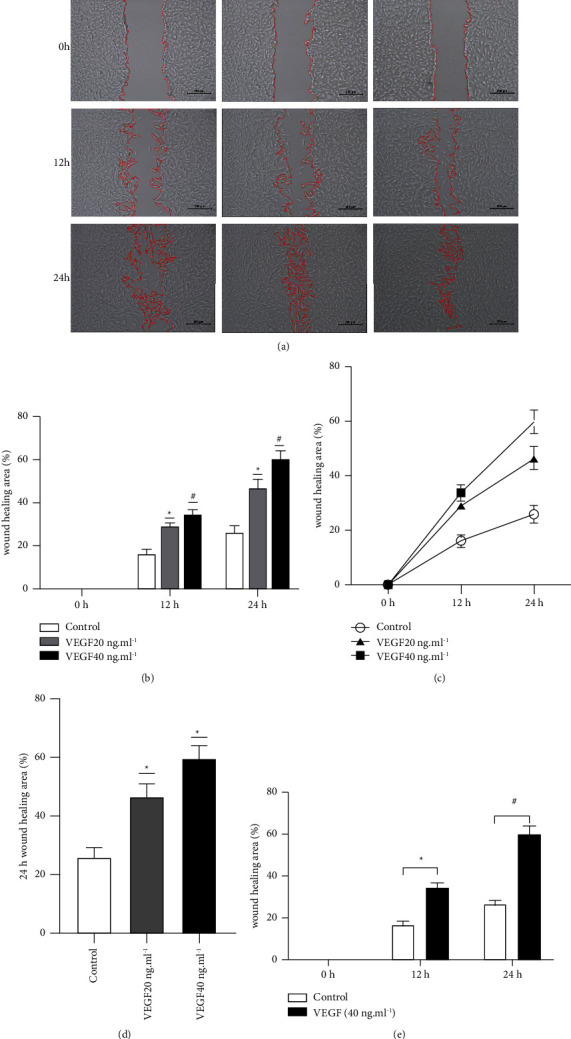
The effect of VEGF on ASMC migration was detected by wound healing assay. (1) The red auxiliary line indicates the scratch healing area of the control group and VEGF (40 ng·ml^−1^) group at 0, 12, and 24 h. (a) A representative picture. (b) Bar graph shows the healing rate of scratches in the control and VEGF groups at 12 and 24 h. (c) Broken line diagram shows the scratch healing rate of the control and VEGF groups at 0, 12, and 24 h, which is statistically significant between the groups (*P* < 0.05). (2) The red auxiliary line is the control, VEGF 20 ng·ml^−1^, and VEGF 40 ng·ml^−1^ groups (24 h). (d) A representative picture. (e) A histogram of the control, VEGF 20 ng·ml^−1^, and VEGF 40 ng·ml^−1^ groups' (24 h) wound healing rate. ^*∗*^*P* < 0.05, ^#^*P* < 0.05; the difference between groups was significant (*P* < 0.05).

To examine the effect of VEGF on ASMC migration, we designed a wound healing assay to observe cell migration. The experiment was divided into a control group, a VEGF 20 ng·ml^−1^ group, and a VEGF 40 ng·ml^−1^ group. According to the wound healing assay, the cell migration rate was 0.162 ± 0.022 at 12 h and 0.260 ± 0.032 at 24 h in the control group; 0.291 ± 0.014 at 12 h and 0.466 ± 0.042 at 24 h in the VEGF 20 ng·ml^−1^ group; and 0.338 ± 0.030 at 12 h and 0.594 ± 0.04 at 24 h in the VEGF 40 ng·ml^−1^ group (Figure 3).

We compared the effects of different concentrations of VEGF on the migration of ASMCs (Figure 3(d)). The 24 h cell migration rates of the VEGF 20 ng·ml^−1^ group were compared with those of the control group, and there were significant differences between the two groups (*P* < 0.05). The 24 h cell migration rates of the VEGF 40 ng·ml^−1^ group were compared with those of the control group, and there were significant differences between the groups (*P* < 0.05). We also compared the effect of VEGF (40 ng·ml^−1^) on the migration of ASMCs at different times (Figure 3(b)). The migration rates of the control group and VEGF (40 ng·ml^−1^) were compared at 12 and 24 h, respectively. The difference between groups was statistically significant (*P* < 0.05). Therefore, the VEGF (40 ng·ml^−1^) group had the most obvious migration trend at 24 h.

### 3.3. Transwell Assay to Detect the Effect of VEGF on Cell Migration

Transwell assays were used to detect VEGF-induced ASMC migration. The experiment was divided into control, VEGF 20 ng·ml^−1^, VEGF 40 ng·ml^−1^, and PDGF 10 ng·ml^−1^ groups. The PDGF 10 ng·ml^−1^ group was used as the positive control group.

After adding VEGF for 12 h, the experiment was terminated. The numbers of migrating cells in the control, VEGF 20 ng·ml^−1^, VEGF 40 ng·ml^−1^, and PDGF 10 ng·ml^−1^ groups were 19.50 ± 7.59, 22.60 ± 3.85, 27.00 ± 3.37, and 81.20 ± 8.90, respectively. After adding VEGF for 24 h, the experiment was terminated. The numbers of migrating cells in the control, VEGF 20 ng·ml^−1^, VEGF 40 ng·ml^−1^, and PDGF 10 ng·ml^−1^ groups were 55.20 ± 6.83, 62.20 ± 6.50, 89.00 ± 4.00, and 118.40 ± 11.91, respectively.

The results showed that after 12 h of VEGF treatment, there was no significant difference in the number of cells that migrated in the VEGF 20 ng·ml^−1^ group compared with the control group (*P* > 0.05). There was no significant difference in the number of cells that migrated in the VEGF 40 ng·ml^−1^ group compared with the control group (*P* > 0.05).

**Figure 4:**
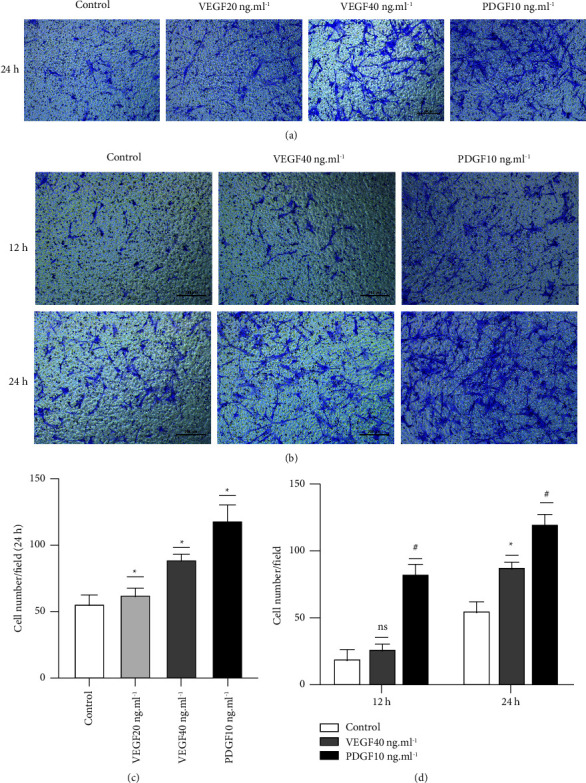
Transwell assays detected the effect of VEGF on ASMC migration. (1) Transwell assays detected VEGF 20 ng·ml^−1^ in the control, VEGF 40 ng·ml^−1^, and PDGF 10 ng·ml^−1^ groups. The effect of group on ASMC migration; PDGF 10 ng·ml^−1^ was used as the positive control group. (a) A representative picture. (b) The histogram shows the number of migrating cells in each group, and the data are expressed as the mean ± standard deviation (M ± SD). (2) Transwell assays were used to detect the effect of VEGF (40 ng·ml^−1^) and the control group on ASMC migration at 12 and 24 h. (c) A representative picture. The histogram in (d) shows the number of cells that migrated in the control and VEGF 40 ng·ml^−1^ groups at 12 and 24 h, and the data are expressed as the mean ± standard deviation (M ± SD). NS indicates that the difference between groups is nonsignificant (*P* > 0.05), ^*∗*^*P* < 0.05, and the difference between groups was significant (*P* < 0.05).

After adding VEGF for 24 h, the migration experiment was terminated. The number of cells that migrated in the VEGF 20 ng·ml^−1^ group was significantly different (*P* < 0.05) from that in the control group. The number of cells that migrated in the VEGF 40 ng·ml^−1^ group was significantly different (*P* < 0.05) from that in the control group (Figure 4).

### 3.4. Asthma Medications Inhibit VEGF-Induced ASMC Migration

**Figure 5:**
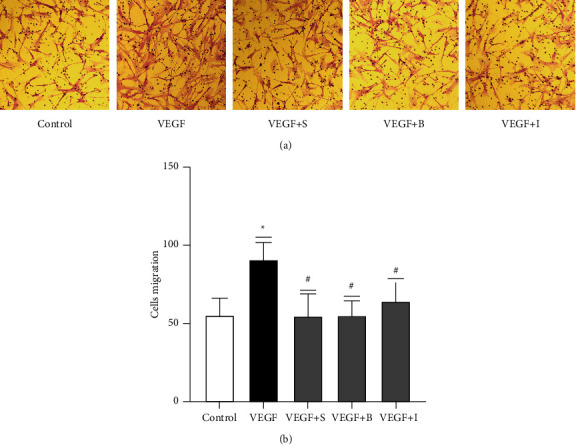
Salbutamol, budesonide, and ipratropium bromide inhibited VEGF-induced ASMC migration. (a) A representative image of cell migration. (b) The number of migrating cells in each group, and the data are expressed as the mean ± standard deviation (M ± SD). ^*∗*^The VEGF group result was significantly different from the control group result (*P* < 0.05). ^#^There are significant differences between the VEGF + S group, VEGF + B group, and VEGF + I group and VEGF group (*P* < 0.05). VEGF + S represents the VEGF + salbutamol group, VEGF + B represents the VEGF + budesonide group, and VEGF + I represents the VEGF + ipratropium bromide group.

We used transwell assays to examine the effects of conventional asthma medications on VEGF-induced ASMC migration (Figure 5). The experiment was divided into control, VEGF (40 ng·ml^−1^), VEGF + salbutamol, VEGF + budesonide, and VEGF + ipratropium bromide groups. The experimental results showed that the numbers of migrating cells in the control, VEGF, VEGF + salbutamol, VEGF + budesonide, and VEGF + ipratropium bromide groups were 55.60 ± 10.14, 91.40 ± 9.52, 54.73 ± 13.88, 55.00 ± 9.91, and 64.27 ± 11.96, respectively. The difference between the VEGF and control groups was statistically significant (*P* < 0.05). Compared with the VEGF group, the VEGF + salbutamol, VEGF + budesonide, and VEGF + ipratropium bromide groups exhibited statistically significant differences (*P* < 0.05). The experimental results showed that adding salbutamol, budesonide, and ipratropium bromide to VEGF significantly reduced the number of cells migrating across the membrane. Therefore, the asthma drugs salbutamol, budesonide, and ipratropium bromide inhibited the VEGF-induced migratory effects of ASMCs (Figure 5).

### 3.5. Effects of Inhibitors of Intracellular Signaling Pathway Targets on VEGF-Induced ASMC Migration

**Figure 6:**
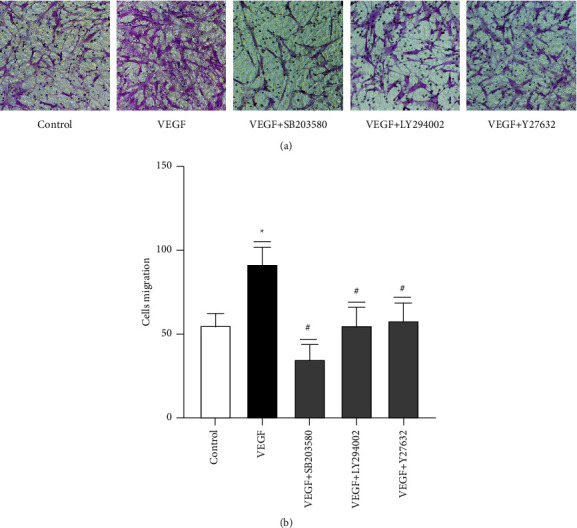
Effect of signaling pathway inhibitors on VEGF-induced ASMC migration. (a) A representative image of cell migration. (b) The histogram shows the number of migrating cells in each group, and the data are expressed as the mean ± standard deviation (M ± SD). ^*∗*^*P* < 0.05. ^#^There are significant differences between the VEGF + SB203580 group, VEGF + LY294002 group, and VEGF + Y27632 group and VEGF group (*P* < 0.05).

We used transwell assays to examine the effects of SB203580, LY294002, and Y27632 on VEGF-induced ASMC migration. The experiment was divided into the control, VEGF (40 ng·ml^−1^), VEGF + SB203580, VEGF + LY294002, and VEGF + Y27632 groups. The experimental results showed that the number of migrating ASMCs at 24 h in the control group was 53.67 ± 8.04, the number of migrating cells in the VEGF group was 91.87 ± 9.37, the number of migrating cells in the VEGF + SB203580 group was 35.13 ± 8.38, and the number of migrating cells in the VEGF + LY294002 group was 55.07 ± 10.51. The number of migrating cells in the VEGF + Y27632 group was 58.07 ± 10.32. The difference between the VEGF and control groups was statistically significant (*P* < 0.05). Compared with the VEGF group, the VEGF + SB203580, VEGF + LY294002, and VEGF + *Y*27632 groups exhibited statistically significant differences (*P* < 0.05). Experiments showed that combining SB203580, LY294002, or Y27632 with VEGF reduced the number of cells migrating across the membrane. Thus, B203580, LY294002, and Y27632 inhibit VEGF-induced ASMC migration (Figure 6).

## 4. Discussion

Asthma is a chronic airway inflammatory disease, and airway remodeling is one of the pathological features of asthma [1]. Abnormal migration of ASMCs is closely related to the pathogenesis of asthma-related airway remodeling [5]. In this study, the effect of VEGF on ASMC migration was investigated by *in vitro* cell experiments to further explore the pathogenesis of airway remodeling in asthma.

VEGF is a potent stimulator of angiogenesis in asthma, and VEGF expression is elevated in the lung tissue and sputum of asthmatic patients [9]. The inhibition of VEGF significantly reduces goblet cell hyperplasia and basement membrane thickening [11]. Lopez-Guisa et al. suggested that airway epithelial cells secrete VEGF, which promotes airway remodeling [12]. Lee et al. proposed that VEGF can induce asthma-like symptoms such as airway inflammation, edema, vascular remodeling, smooth muscle cell proliferation, and airway hyperresponsiveness through IL-13-dependent and IL-13-independent pathways [13]. In this study, both scratch and transwell assays demonstrated that VEGF induction enhanced the migration of ASMCs. Studying the regulatory mechanism of VEGF on ASMC migration has potential clinical value. Early intervention in ASMC migration can prevent airway remodeling. Therefore, in this study, different asthma drugs and signaling pathway inhibitors were used to interfere with ASMC migration to screen for effective drugs. This study provides the basis for the treatment of airway remodeling.

Salbutamol is a short-acting *β*2-adrenergic receptor agonist and a commonly used drug for the treatment of asthma. It can effectively inhibit the release of allergic substances such as histamine and inhibit the migration of ASMCs [13]. In the pathogenesis of asthma, inflammatory mediators can increase the migration of ASMCs; however, glucocorticoids (GCs) are the gold standard for treating asthmatic airway inflammation and can effectively reduce asthmatic airway inflammation. Budesonide is a glucocorticoid with a highly effective local anti-inflammatory effect that is commonly used in treating acute asthma attacks by inhalation. In addition to its good anti-inflammatory effect, budesonide can also stimulate the rapid production of cAMP by ASMCs, which enhances the small airway bronchodilation induced by adrenergic receptor agonists [14]. Sun et al. reported that glucocorticoids can downregulate the expression of VEGF to inhibit angiogenesis in airway remodeling [15]. Ipratropium bromide is an M-receptor anticholinergic drug that acts on M-receptors on the surface of smooth muscle and inhibits the abnormal proliferation and migration of ASMCs by selectively blocking the binding of acetylcholine to M-receptors, thereby reducing airway remodeling [16]. Eckard Hamelmann et al. showed that ipratropium bromide can reduce vagus nerve tension, reduce intracellular calcium ion concentration, and play a role in bronchial relaxation; furthermore, it can inhibit the excessive secretion of airway glands, reduce airway hyperactivity, increase responsiveness, and improve lung function [17]. Short-acting *β*2-adrenergic receptor agonists, glucocorticoids, and M-receptor anticholinergics are widely used in traditional asthma treatment, improving the clinical symptoms of asthma patients through different mechanisms.

Our study showed that salbutamol, budesonide, and ipratropium bromide inhibited VEGF-induced ASMC migration and have clinical value in treating airway remodeling. Beta2-adrenergic agonists and cortisol hormone therapy are common and classic strategies for treating bronchial asthma, although this therapy provides rapid relief of acute asthma symptoms and reduction of bronchoconstriction and airway inflammation in most patients [17]. However, some patients are not sensitive to common and classical treatments [18]. Alternatively, low compliance or adverse side effects may occur due to long-term continuous use [19, 20]. Therefore, it is necessary to develop new drugs to treat asthma-related airway remodeling and find feasible alternative therapies. Signaling pathway inhibitor drugs may be a new way to treat asthma-related airway remodeling in the future.

Salter et al. recently identified various potential intracellular targets for the inhibition of ASMC migration, including MAPK, Src family tyrosine kinase, PI3K, and Rho-b kinase inhibitors [21]. Studies have reported changes in kinase activation status in all asthma patients, especially in severe asthma patients, the responsiveness to glucocorticoids is reduced, and the changes in kinase activation status are more obvious [22]. Some researchers believe that extracellular signal-related kinase (ERK) and p38MAPK, among others, play a role in steroid-insensitive asthma patients in a stimulus-dependent manner [23, 24]. P38MAPK is involved in the inflammatory process and airway remodeling of the lower airways. Patients with severe asthma are insensitive to cortisol, which can be improved by combined treatment with p38MAPK inhibitors and dexamethasone [25]. In the transwell experiments in this study, VEGF-induced ASMC migration was inhibited after ASMCs were pretreated with SB203580.

PI3K is a chemotactic signal amplifier that accumulates at the leading edge of migrating cells [26]. In ASMCs, the PI3K pathway has been identified as a key signaling mechanism in balancing the promigratory and antimigratory signals of Rho-guanosine triphosphate (GTPase) activating protein (GAP) [21, 27]. LY294002 is a broad-spectrum PI3K inhibitor that reversibly binds the kinase domain of DNA-PK. LY294002 inhibited VEGF-induced ASMC migration in the transwell assay; therefore, PI3K may regulate the key target of VEGF-induced ASMC migration.

Y-27632 is an inhibitor of ROCK, a downstream effector of RhoA. It can bind to the catalytic site of ROCK to inhibit kinase activity and reduce the expression levels of F-actin and ɑ-tubulin in ASMCs, suggesting that Rho kinase is involved in ASMCs with asthmatic airway remodeling. The cytoskeleton is tightly connected [28]. As a small molecule protein of the Ras family, RhoA regulates cytoskeletal proteins and promotes cell migration. Activated RhoA activates its downstream ROCK, phosphorylates and inactivates myosin phosphatase, resulting in the inability of phosphorylated myosin to dephosphorylate, increases the level of phosphorylated myosin in the cytoplasm, promotes the interaction between actin and myosin, and is conducive to cell contraction and migration [29]. The Rho/Rho kinase pathway is involved in the directed and undirected cell migration of ASMCs by regulating actin cytoskeleton rearrangement [30, 31]. VEGF-induced ASMC migration was attenuated after pretreatment of ASMCs with Y-27632, suggesting that RhoA/ROCK signaling may be involved in VEGF-induced ASMC migration.

Inhibitors B203580, LY294002, and Y27632 inhibit VEGF-induced ASMC migration through different intracellular targets. Therefore, regulating the key targets of the p38 MAPK, PI3K, and ROCK signaling pathways can effectively regulate VEGF-induced ASMC migration to inhibit airway remodeling.

These findings provide new insights that VEGF-induced enhancement of ASMC migration, the current traditional drugs budesonide, terbutaline, and ipratropium bromide can inhibit this migration, the p38 MAPK, PI3K, and ROCK signal pathway target inhibitors may be alternative to traditional drugs in the future. Of course, the related mechanism and safety profile still need further research.

## Data Availability

The data used to support the findings of this study are available from the corresponding author upon request.

## Abbreviations

ASMCs: Airway smooth muscle cells
VEGF: Vascular endothelial growth factor
PDGF: Platelet-derived growth factor
GC: Glucocorticoids
ERK: Extracellular signal-related kinase
p38MAPK: p38 mitogen-activated protein kinase.
